# Advanced and Stable Metal-Free Electrocatalyst for Energy Storage and Conversion: The Structure–Effect Relationship of Heteroatoms in Carbon

**DOI:** 10.1021/acsomega.3c01145

**Published:** 2023-05-01

**Authors:** Jingjing Zhang, Kechuang Wan, Ping Wen Ming, Bing Li, Cunman Zhang

**Affiliations:** Clean Energy Automotive Engineering Center and School of Automotive Studies, Tongji University, Shanghai 201804, China

## Abstract

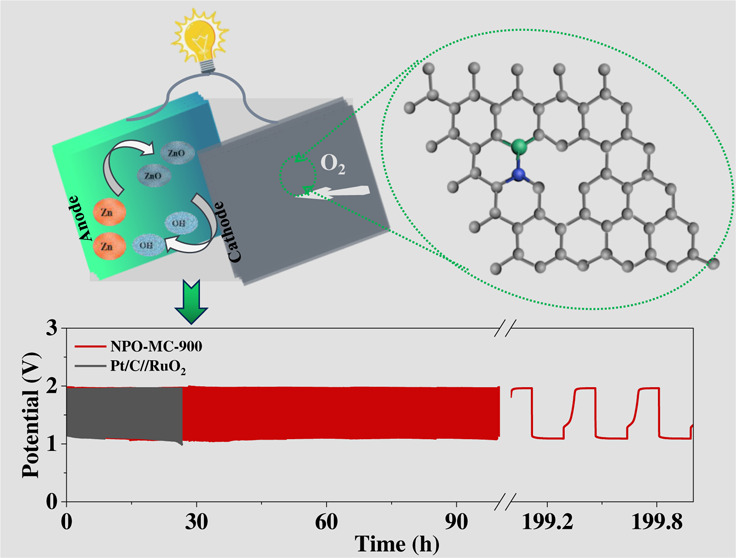

Ever-developing energy device technologies require the exploration of advanced materials with multiple functions. Heteroatom-doped carbon has been attracting attention as an advanced electrocatalyst for zinc–air fuel cell applications. However, the efficient use of heteroatoms and the identification of active sites are still worth investigating. Herein, a tridoped carbon is designed in this work with multiple porosities and high specific surface area (980 m^–2^ g^–1^). The synergistic effects of nitrogen (N), phosphorus (P), and oxygen (O) in micromesoporous carbon on oxygen reduction reaction (ORR)/oxygen evolution reaction (OER) catalysis are first investigated comprehensively. Metal-free N-, P-, and O-codoped micromesoporous carbon (NPO-MC) exhibits attractive catalytic activity in zinc–air batteries and outperforms a number of other catalysts. Combined with a detailed study of N, P, and O dopants, four optimized doped carbon structures are employed. Meanwhile, density functional theory (DFT) calculations are made for the codoped species. The lowest free energy barrier for the ORR can be attributed to the pyridine nitrogen and N–P doping structures, which is an important reason for the remarkable performance of NPO-MC catalyst in electrocatalysis.

## 1 Introduction

Rechargeable zinc–air batteries (ZABs) are a promising portable energy device due to their high energy density, high safety, and environmental friendliness, and the high theoretical specific energy of ZABs up to 1218 Wh kg^–1^ is far more than that of lithium–sulfur batteries.^1−5^ However, it seriously hinders its overall performance due to the slow kinetic reaction of the oxygen reduction reaction (ORR) and the oxygen evolution reaction (OER) that occurs at the cathode of ZABs;^6−8^ therefore, the development of highly efficient ORR and OER catalysts is urgent. At present, precious metals (Pt, RuO_2_, etc.) are used as high-efficiency catalysts to increase the kinetic reaction rate; however, the high cost and resource shortage limit the further commercial application of precious metal catalysts. This prompted us to seek low-cost, resource-rich, efficient, and stable catalysts.^9−12^ Carbon materials of metal-free heteroatom doping are among the most promising alternatives to precious metal catalyst materials due to their competitive catalytic activity, cost-efficiency, abundant resources, and satisfactory conductivity.^13−15^ The competitive electrocatalytic activity benefits from the abundance of active sites generated by the doping of heteroatoms with different electronegativities and atomic radii.^16−19^ For example, in nitrogen-doped carbon, more positive charges are generated due to the lower electronegativity of carbon than that of nitrogen and the charge transfer between carbon and adjacent heteroatoms, which facilely causes the adsorption of O_2_.^20−22^ However, the incorporation of P can provide a defect-induced active surface that is prone to adsorption of oxygen, leading to a further decrease in the free energy of the reaction.^23−25^ As for the introduction of O, the O atoms can readily bond with the carbon of sp^2^ hybridization, thereby modulating intrinsic electrons, increasing the surface area of the carbon matrix and making the material rich in active sites.^26−28^ However, the electrocatalytic activity of multidoped carbon materials is largely better than that of single-doped carbon, which may be because the synergetic coupling effect between different dopants significantly improves the bifunctional catalytic activity compared with the activity of single-doped carbon with insufficient sites. For example, (i) it is difficult for P to be successfully doped due to the large atomic radius.^29−32^ (ii) Guo et al.^33^ prepared N,P-codoped carbon (PNGF_DAP_800) and confirmed that its high-temperature carbonized sample PNGF_DAP_800 had much lower OER activity due to the removal of thermally unstable P–N bonds. Therefore, it is important to prepare materials with stable N–P structures and rich N and P elements. Hexachlorocyclotriphosphazene (HCCP) has been favored by researchers because of its rich N and P elements.^34,35^ In the past, HCCP was mostly used in antitumor drugs and flame-retardant cotton.^36^ In addition, HCCP itself is rich in N–P structures, which can form N,P-codoped carbon materials in situ during the pyrolysis process, and its own N–P structure remains stable in high-temperature carbonization.^37^ Although heteroatom doping has many advantages, it is restricted by chemical reaction steps, which is not conducive to the wide application of heteroatom doping materials. Xiao et al.^38^ prepared hierarchically porous nitrogen-doped carbon (HPNC) by a silica template method and zinc nitrate. The template method for synthesizing nanomaterials generally has removal problems. Tian et al.^39^ prepared a nitrogen-doped carbon network (F/P–N–C-950) through an efficient strategy. However, it has a maximum power density of only 138 mW cm^–2^, which is not sufficient to compete with other same-class materials. Therefore, it is particularly important to develop high-performance materials that are simple to synthesize and rich in heteroatoms.

Here, we prepare a new cross-linked structure, using heteroatom-rich HCCP as the nitrogen and phosphorus source, and polymerize with poly(ethyleneimine) in one step at room temperature. The main feature of this method is that the activity of PEI is reduced, which controls the polymerization rate of PEI and HCCP, obtaining a highly homogeneous product. In addition, the steric hindrance of PEI is small, the crosslinking between PEI and HCCP is more than sufficient, and a stabilizing polymeric structure is obtained. This multidoped carbon material catalyst (NPO-MC) was obtained by high-temperature carbonization. NPO-MC exhibits a half-slope potential equivalent to that of commercial platinum carbon. The high specific surface area and good electrical conductivity endow NPO-MC-900 with efficient power density (215 mW cm^–2^) and cycle performance (>200 h). At the same time, the active site of NPO-MC has also been confirmed by density functional theory. The activation energies of the pyri-*N* and N–P models were the lowest, with the fastest kinetic response, which improves the catalytic activity of NPO-MC. Our work provides a reference for the preparation of metal-free heteroatom doping for application in the energy field.

## 2 Experimental Section

### 2.1 Synthesis of NPO-MC

The NPO-MC was synthesized as follows: First, PEI (1.95 g, 0.033 mol) and triethylamine (TEA) (1.55 g, 0.015 mol) were dissolved in acetonitrile (50 mL), followed by ultrasonication for 0.5 h. Subsequently, HCCP (0.00125 mol) was dissolved in acetonitrile (20 mL), followed by ultrasonication at room temperature for 0.5 h. This was dropped into the above solution. The polycondensation reaction was carried out in an ultrasonic bath under the same conditions for another 4 h. Second, a light yellow precipitate was produced in the above solution; the samples were collected by filtration and washed three times using ethanol and deionized water (1000 mL) consecutively. HCCP//PEI materials were finally obtained after vacuum drying. Third, the pyrolyzed composites were thermally treated under different carbonation temperatures (800, 900, and 1000 °C for 3 h under a N_2_ atmosphere), and different amounts of monomer (HCCP: 0.000625, 0.00125, and 0.0025 mol) were added. Further details on the properties of material are provided in the Supporting Information.

### 2.2 Material Characterization

Please see the detailed processes in the Supporting Information.

### 2.3 Electrochemical Measurements

A typical three-electrode system was set up. Please see the detailed processes in the Supporting Information.

### 2.4 Zn–Air Battery Assembly

In order to evaluate the performance of these catalysts, a homemade zinc–air cell was assembled. Details of the procedure are provided in the Supporting Information.

### 2.5 Computational Details

For more information on density functional theory (DFT) calculations, please see the Supporting Information.

## 3 Results and Discussion

### 3.1 Characterization of NPO-MC

**Figure 1:**
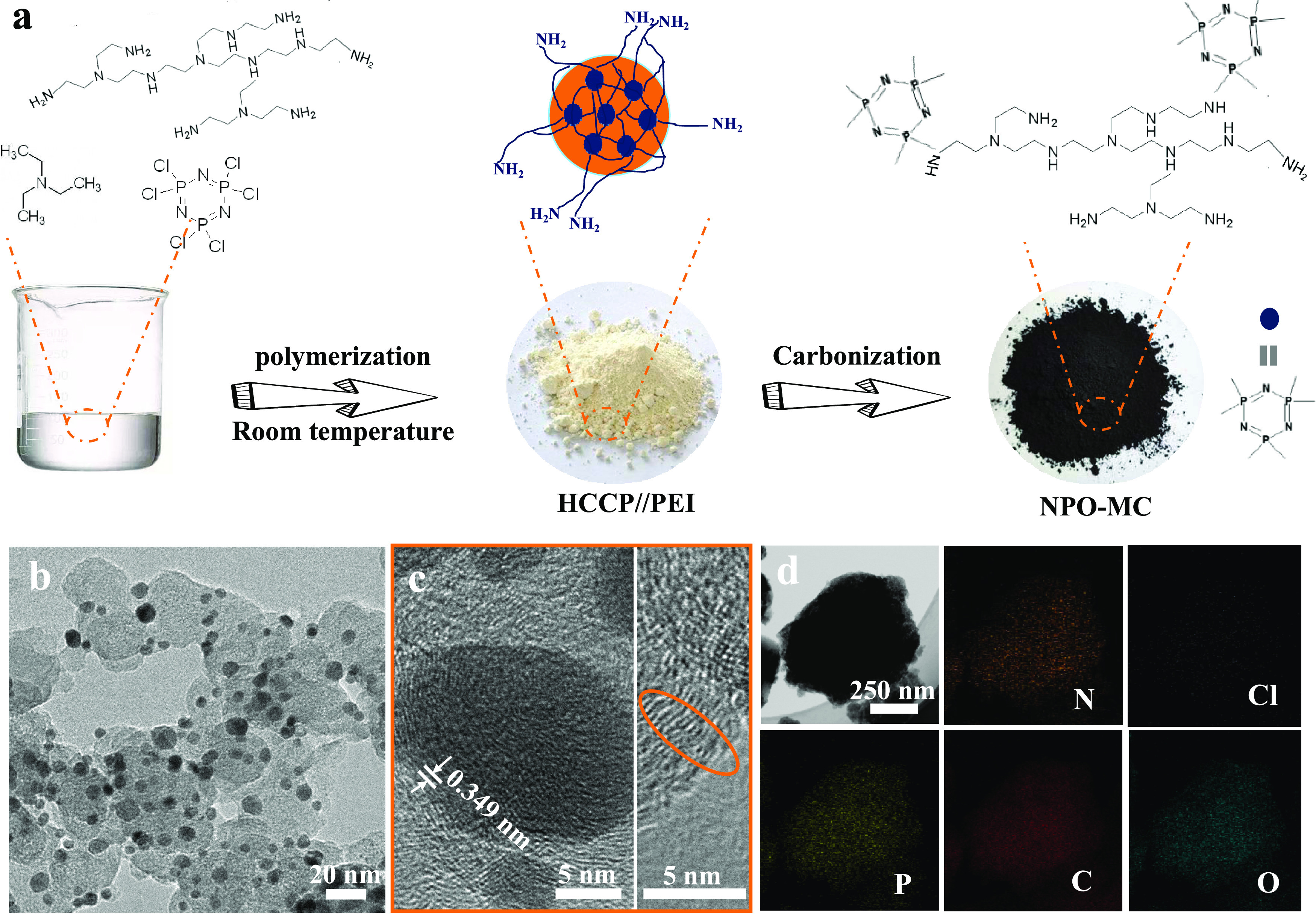
Morphological characterization of NPO-MC-900-0.00125. (a) Synthetic route of NPO-MC; (b) TEM image; (c) HRTEM images; and (d) elemental mapping of N, O, P, and C in NPO-MC.

Figure 1a schematically illustrates the synthetic procedure of NPO-MC. HCCP and PEI were used to produce light yellow oligomers (N,P,O-codoped carbon) by a polycondensation reaction under the regulation of acid-binding agent TEA. The HCl byproduct produced during the reaction was removed by TEA, followed by carbonization under a nitrogen atmosphere. The amount of HCCP was adjusted to control the content of N and P. The amount of HCCP in NPO-MC-0.000625, NPO-MC-0.0025, and NPO-MC-0.00125 was 0.000625, 0.0025, and 0.00125 mol, respectively. The effect of monomer concentration on the formation of NPO-MC was investigated (Figure S1a–c in the Supporting Information). With an increase of HCCP amount from 0.000625 to 0.0025 mol, the product gradually changes from cross-linked to a dispersed spherical shape, and with a further increase of the HCCP amount, products with aggregated spherical morphology were obtained; this may be caused by an excess of HCCP. Figure 1b shows the morphology of heteroatom-doped carbon NPO-MC-0.00125-900. The high-resolution transmission electron microscopy (HRTEM) image presented a dispersed spherical state; the dark-colored spherical part is a solid structure formed by the aggregation of small particles, which should be a highly cross-linked structure with phosphonitrile as the matrix. The HRTEM image further illustrates the graphitized shells with multichannel properties (orange circle in Figure 1c), where the porous nature of the graphitized shells can further remedy the mass transfer problem of the nanomaterials. NPO-MC-900 exhibits distinct lattice edges (Figure 1c) due to heteroatom doping and reduced defect levels under high-temperature carbonization. Furthermore, the detailed elemental information of the NPO-MC-900 sample obtained by high-angle annular dark-field scanning transmission electron microscopy (HAADF-STEM) was used (Figure 1d). The successful introduction of N, O, and P was confirmed by the uniform distribution mapping of the elements, in which unexpected oxygen element doping occurred during the washing step and calcination processes. As expected, the elemental chlorine has been barely captured by the binding of HCl products to TEA and the volatilization of HCl gas by high-temperature calcination, indicating that the N, O, and P codoped carbon.

**Figure 2:**
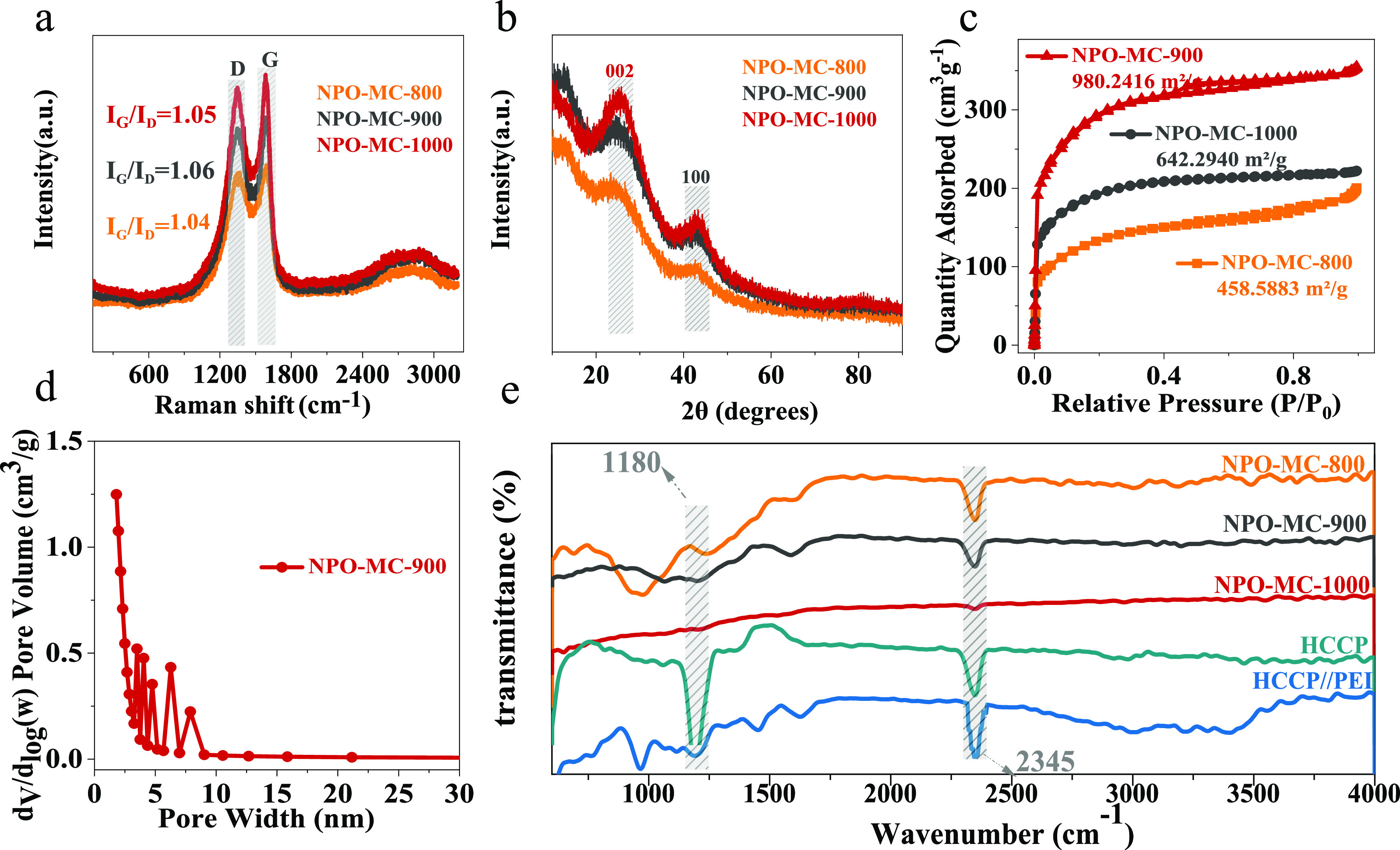
Structural characterization of NPO-MC-800, NPO-MC-900, and NPO-MC-1000 samples. (a) Raman spectra; (b) XRD spectra; (c) N_2_ adsorption–desorption isotherm; (d) pore distribution curve; (e) FTIR spectra of NPO-MC-800, NPO-MC-900, and NPO-MC-1000 samples and HCCP and HCCP//PEI reference sample.

The Raman spectra of NPO-MC are shown in Figure S2 in the Supporting Information. The D-band (≈1361 cm^–1^) and G-band (≈1579 cm^–1^) correspond to a broad disorder induction and an in-plane vibration, respectively. The high graphitization of NPO-MC is reflected in the *I*_G_/*I*_D_ ratio, and Figure 2a shows the Raman spectra at different carbonization temperatures. The pyrolysis temperature of 1000 and 900 °C corresponds to the *I*_G_/*I*_D_ value (1.05) and (1.06), respectively. The higher graphitization of materials was deemed to reveal higher conductivity, which would increase the charge transport during electrochemical reactions. The X-ray diffraction (XRD) patterns of NPO-MC-800, NPO-MC-900, and NPO-MC-1000 are shown in Figure 2b; this shows that all materials have two peaks around 25 and 43°, which are attributed to the graphitized (002) and (100) diffraction planes. The XRD patterns of NPO-MC-0.000625, NPO-MC-0.00125, and NPO-MC-0.0025 are presented in Figure S3 in the Supporting Information. The pore distribution feature of NPO-MC samples was analyzed by N_2_ adsorption isotherms (in Figure 2c). Based on the calculations, the specific surface areas of NPO-MC-800, NPO-MC-900, and NPO-MC-1000 were 458, 980, and 642 m^2^ g^–1^, respectively. Meanwhile, Figure 2d shows the pore size distribution of the sample, which belongs to the mesoporous zone (pore sizes are mainly distributed in the range of 2–10 nm). This nanoporous structure promotes the migration and infiltration of electrolytes into the electrode, and electrochemical reaction kinetics are accelerated. As a comparison, the Brunauer–Emmett–Teller (BET) measurements for NPO-MC-0.0065/0.00125/0.0025-900 samples were also obtained (Figure S4 in the Supporting Information) and were 745, 980, and 584 m^2^ g^–1^, respectively. After the incorporation of N, P, and O, the surface area greatly improved. Abundant active sites and fast mass transport benefit from the large surface area and abundant nanopores.^40,41^Figure S5 in the Supporting Information shows the Nyquist plots of NPO-MC catalysts in 0.1 M KOH. Obviously, all three curves are composed of a semicircle at the high frequency and a straight line at the low frequency. As indicated in the Nyquist plots of NPO-MC-900 compared with NPO-MC-800 and NPO-MC-1000 given in Figure S5, the charge transfer resistance (*R*_ct_) of NPO-MC-900 (38 Ω) was much smaller than other samples, indicating a faster ORR kinetics of NPO-MC-900. Fourier transform infrared (FTIR) spectra are adopted to characterize the surface chemical structure of the materials (Figure 2e). For the PEI/HCCP, HCCP, NPO-MC-800, and NPO-MC-900 materials, the spectra were normalized to the vibration of the endocyclic P–N bonds (1180 cm^–1^); this is consistent with the data reported earlier.^42^ When the carbonization temperature is increased to 1000 °C, some of the P–N structure may be lost, and as a result, the characteristic peak intensity of P–N is much lower.

**Figure 3:**
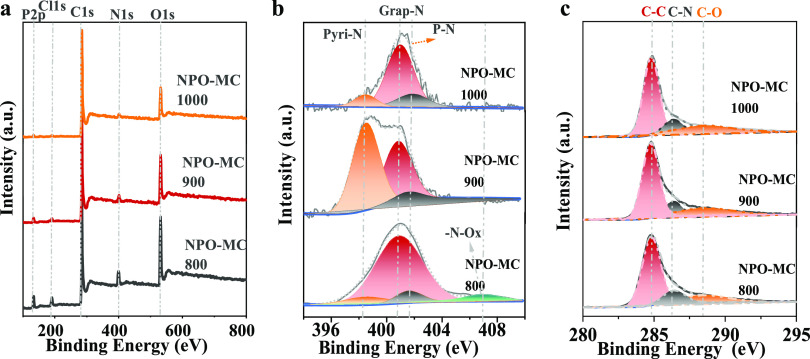
Structural characterization of NPO-MC-800, NPO-MC-900, and NPO-MC-1000 samples. (a) XPS spectra; (b) N 1s spectra; and (c) C 1s spectra.

The elemental compositions and elemental chemical states of the NPO-MC-800, NPO-MC-900, and NPO-MC-1000 samples were further tested by X-ray photoelectron spectroscopy (XPS). The survey spectra (Figure 3a) show the presence of C, N, O, and P in the materials. From Table S1, it can be seen that the chlorine content of the three is relatively low (<0.01%); it can be explained that the loss of Cl element is large at a higher carbonization temperature. Therefore, the effect of chlorine is negligible. The N 1s spectrum of the homemade samples can be divided into three/four types (Figure 3b); among them, the three catalysts are rich in graphitized carbon, which promotes the catalyst to have good electrical conductivity. Pyridinic-*N* bonds and P–N bonds were included in the three catalysts, but N–O*_x_* species existed in NPO-MC-800 material. Table S1 shows that the contents of N, O, and P in NPO-MC-800, NPO-MC-900, and NPO-MC-1000 samples gradually decreased, indicating that the loss of N, O, and P was more severe at higher temperatures.

Figure 3c shows the spectra of C 1s. The C 1s spectra of NPO-MC mainly have three peaks C–C, C–N, and C–O, which are located at 285.19, 286.5, and 288.86 eV,^43,44^ respectively. Figure S6 and Table S1 in the Supporting Information show the oxidation state spectrum of NPO-MC. The proportion of O element content gradually decreases, being 18.7% in NPO-MC-800, 15.8% in NPO-MC-900, and 12.2% in NPO-MC-1000. Meanwhile, the spectrum of O 1s is mainly classified as carbonyl groups (531.1 ± 0.1 eV), N–O–C ether groups (532. 4 ± 0.1 eV), and carboxyl groups (533.6 ± 0.1 eV). Figure S7 in the Supporting Information shows the phosphorus state survey spectra of NPO-MC; the P 2p peaks of NPO-MC are as follows: P–C at 132.3 eV, P–N at 133.7 eV, and P–O at 134.4 eV. From the above material characterization, the successful incorporation of N, O, and P has been confirmed, which is expected to make corresponding contributions to the subsequent electrochemical performance.

**Figure 4:**
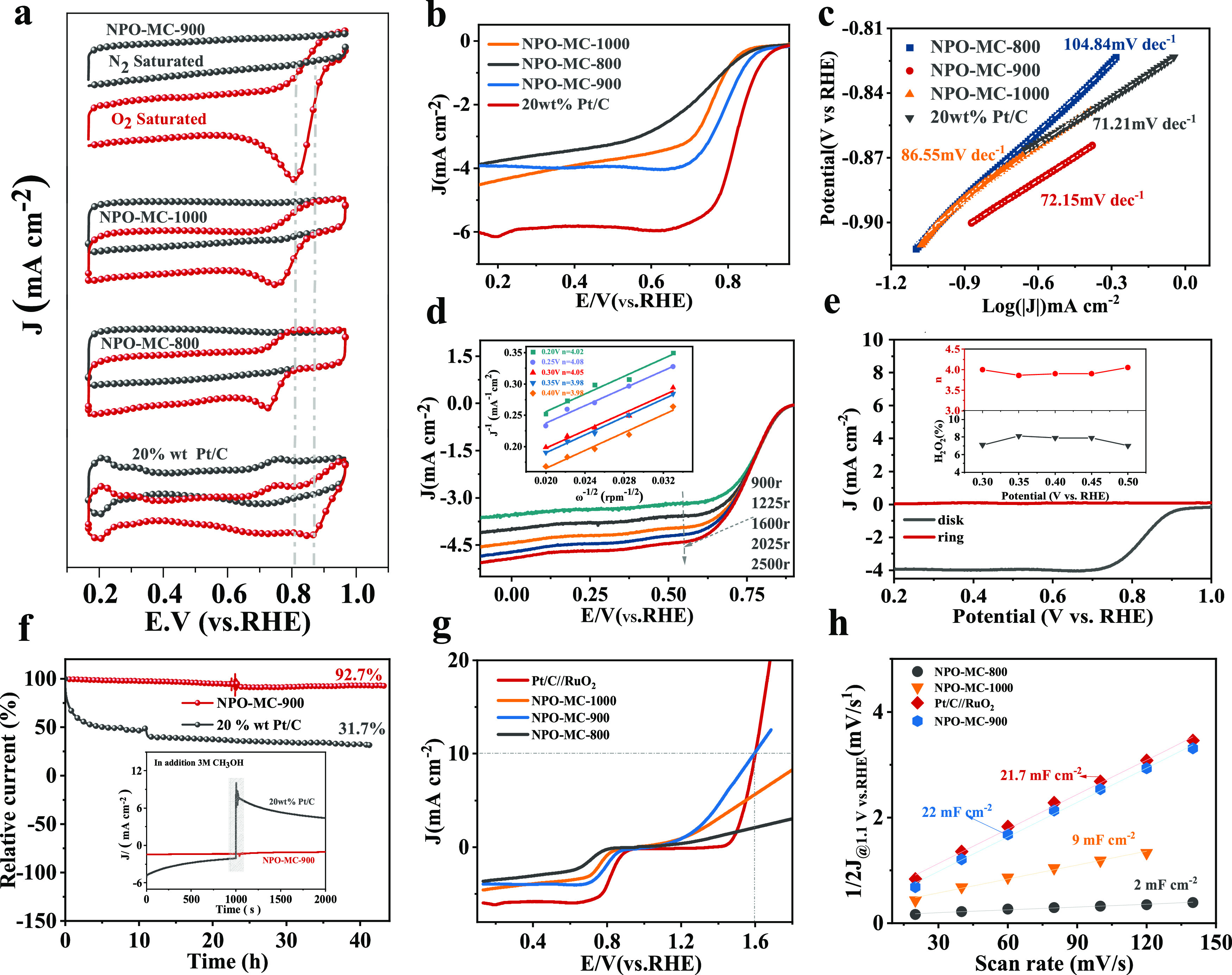
Performance characterization in 0.1 M KOH electrolyte. (a) CV curves for N_2_ saturation (black) and O_2_ saturation (red); (b) linear sweep voltammetry (LSV) curve of 5 mV s^–1^. (c) Tafel diagrams of NPO-MC-800, NPO-MC-900, NPO-MC-1000, and 20 wt % Pt/C catalysts. (d) LSV curve figure of NPO-C-900 speed change at 900–2500 rpm and Koutecky–Levich (K–L) (illustration). (e) RRDE measurements, the calculated H_2_O_2_ selectivity, and electron transfer number (*n*) (inset) of NPO-C-900. (f) *i*–*t* chronoamperometric responses and (inset) methanol tolerances for NPO-C-900 and 20 wt % Pt/C. (g) Comparison of the ORR and OER performance of three catalysts. (h) Linear fitting of scan rates with capacitive current densities for the prepared catalysts.

The electrocatalytic ORR performance of NPO-MC was researched by cyclic voltammetry (CV) in an O_2_-saturated and N_2_-saturated electrolyte 0.1 M KOH solution at a scan rate of 10 mV s^–1^ (Figure 4a). A commercial 20 wt % Pt/C, NPO-MC-800, NPO-C-M900, and NPO-MC-1000 were compared. Potentials were referenced to the reversible hydrogen electrode (RHE). The homemade material showed a distinct reduction peak in the O_2_-saturated electrolyte (pink line). However, there was no oxidation peak in the N_2_-saturated (blue line) solution. All of the NPO-MC-0.00125 materials prepared at 800–1000 °C and the commercialized 20 wt % Pt/C have catalytic activity, and the samples with different high-temperature carbonization have obvious redox peaks. Among them, when the carbonization temperature is 900 °C (NPO-MC-0.00125-900), the position of the redox peak is positively shifted, which is closer to the commercial platinum carbon, indicating that the studied materials have the highest electrocatalytic activity.

### 3.2 Catalytic Performance of NPO-MC

Based on the electrochemical characterization of LSV at 1600 rpm (Figure 4b), the onset potential of NPO-MC-900 was 0.91 V vs RHE and the half-wave potential was 0.79 V vs RHE, which was comparable to the commercial 20 wt % Pt/C (*E*_onset_ is 0.94V vs RHE, *E*_1/2_ is 0.82V vs RHE). The NPO-MC-900 catalyst offers certain advantages over other previously reported metal-free or even metal-containing electrocatalysts (Table S2 in the Supporting Information). The fast and slow ORR kinetics can be evaluated by the Tafel slope (Figure 4c), where the smaller the Tafel slope value, the faster the current density increases, indicating that the catalyst has faster kinetics and better catalytic activity. The Tafel slopes of NPO-MC-800, NPO-MC-900, NPO-MC-1000, and 20% Pt/C are 104.8, 72.1, 86.5, and 71.2 mV dec^–1^, respectively, indicating that both NPO-MC-900 and 20% Pt/C have fast kinetics, which can be attributed to the effect of heteroatom doping, thus improving the electrochemical performance of the ORR. Figure 4d displays the corresponding Koutecky–Levich plots (with RHE) of NPO-MC-900 at rotational speeds of 900r, 1225r, 1600r, 2025r, and 2500r and different potentials. The electron transfer number (*n*) of NPO-MC-900 is approximately equal to 4.0, suggesting that the ORR processes occurring on the catalyst were dominant by a desirable four-electron pathway with negligible formation of peroxide intermediates. Figure 4e shows the H_2_O_2_ measurement of NPO-MC-900 in 0.1 M HClO_4_ with an electron transfer number (*n*) of 3.98–4 and less than 8% of H_2_O_2_ yield (inset Figure 4e), validating the results of Figure 4d; it is further confirmed that NPO-MC-900 is inclined to the four-electron ORR reaction pathway in 0.1 M HClO_4_. Figure 4f reveals the excellent durability of NPO-MC-900, with a current retention of 92.7% after 45 h of cycling, while only 31.7% of 20 wt % Pt/C remains. In addition, NPO-MC-900 showed a high degree of resistance to methanol cross-effects. After injecting methanol into the electrolyte, obvious changes occurred in the Pt/C catalyst, and the reaction of the NPO-MC-900 can be neglected (inset Figure 4f). In summary, NPO-MC-900 is a good metal-free bifunctional catalyst, as shown in Figure 4g. Compared to NPO-MC-800 and NPO-MC-1000 catalysts, the ORR and OER (LSV curves with *iR* correction) performance of NPO-MC-900 is obviously superior, and its OER performance is comparable to commercial Pt/C//RuO_2_. The NPO-MC-900 was also superior in the electrocatalytically active surface area (ECSA), shown in Figure 4h, as investigated via double-layer capacitance (*C*_dl_) and CV measurements (Figure S8 in the Supporting Information).

### 3.3 Zinc–Air Battery

**Figure 5:**
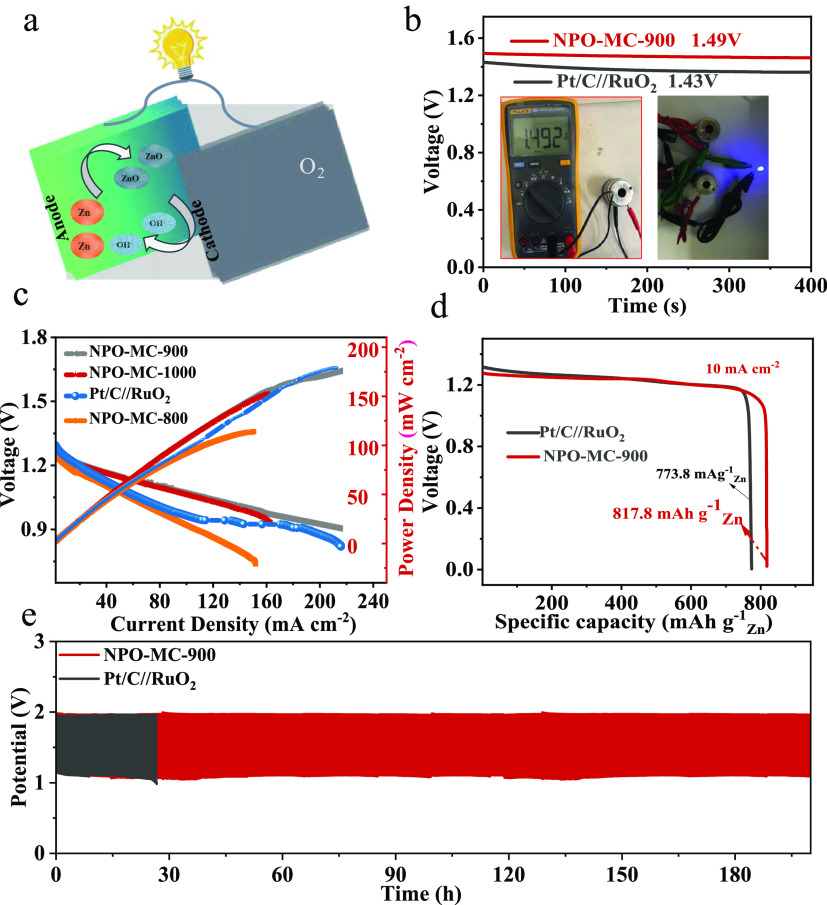
Zn–air battery performance of the NPO-MC-900 catalysts. (a) Diagram of a zinc–air battery; (b) open-circuit plots (inset: photos of LED); (c) discharged curves and power densities of the NPO-MC; (d) specific capacity plots at 10 mA cm^–2^; (e) charge and discharge stability of the Zn–air batteries at a current density of 10 mA cm^–2^.

The outstanding bifunctional properties of NPO-MC-900 were evaluated through zinc–air battery (ZAB) applications. The NPO-MC-900 material was used as the cathode of the battery, and the polished zinc sheet was assembled as the anode, testing in a 6.0 M KOH electrolyte containing 0.2 M Zn (Ac)_2_ (Figure 5a). The open-circuit voltage (OCV) of ZAB with NPO-MC-900 and Pt/C//RuO_2_ as air cathodes is revealed in Figure 5b, which was stabled at 1.49 and 1.43 V, respectively. ZAB with NPO-MC-900 as a bifunctional catalyst is shown in the inset of Figure 5b; to yield a voltage of 1.49 V, two ZABs with NPO-MC materials as cathodes are connected in series to make light-emitting diodes (LEDs) emit light, which is expected to be used in the energy field for NPO-MC materials. The maximum power density of the NPO-MC-900 achieved is 215 mW cm^–2^ (Figure 5c), which is comparable to commercial Pt/C/RuO_2_ performance and exceeds that of other metal catalysts recently reported, e.g., NFPC-1100 porous materials (157 mW cm^–2^),^45^ Co/Co–N–C nanosheets (132 mW cm^–2^),^46^ ball-like CoFe@NOC (205 mW cm^–2^),^47^ porous N,B-codoped carbon nanotubes (NBCNTs, 173.9 mW cm^–2^),^48^ hierarchical porous carbon nanoshells (NPS-HPCNs, 206 mW cm^–2^),^49^ and jagged carbon nanotubes (JCNTs, 142 mW cm^–2^).^50^ The above results indicate that NPO-MC-900 displays excellent performance in the Zn–air cell. This is in agreement with the characterization and electrochemical test results. More active sites are provided due to the pyri-*N*, N–P, and large specific surface area of NPO-MC-900. Meanwhile, the abundant micromesopores in NPO-MC-900 provide a favorable channel for mass transfer. The NPO-MC-900 provides a relatively stable discharge platform with only slight degradation (Figure 5d). The specific discharge capacity is estimated to be 817 mAh g_Zn_^–1^ at 10 mA cm^–2^, which is higher than that of Pt/C//RuO_2_ (773 mAh g_Zn_^–1^). The excellent performance of NPO-C-0.00125-900 in Zn–air batteries indicates its great potential in practical application, further indicating the excellent performance of NPO-MC-900 in Zn–air batteries. Meanwhile, the long-term stability tests for the batteries assembled with NPO-MC-900 were evaluated by alternating a 10 min discharging process and a 10 min charging process continuously at 10 mA cm^–2^ and cycled for more than 200 h without significant degradation; however, the cells assembled with Pt/C/RuO_2_ cycled for 30 h under the same conditions already showed significant degradation, indicating the excellent stability of NPO-MC-900 (Figure 5e).

### 3.4 DFT Computation

**Figure 6:**
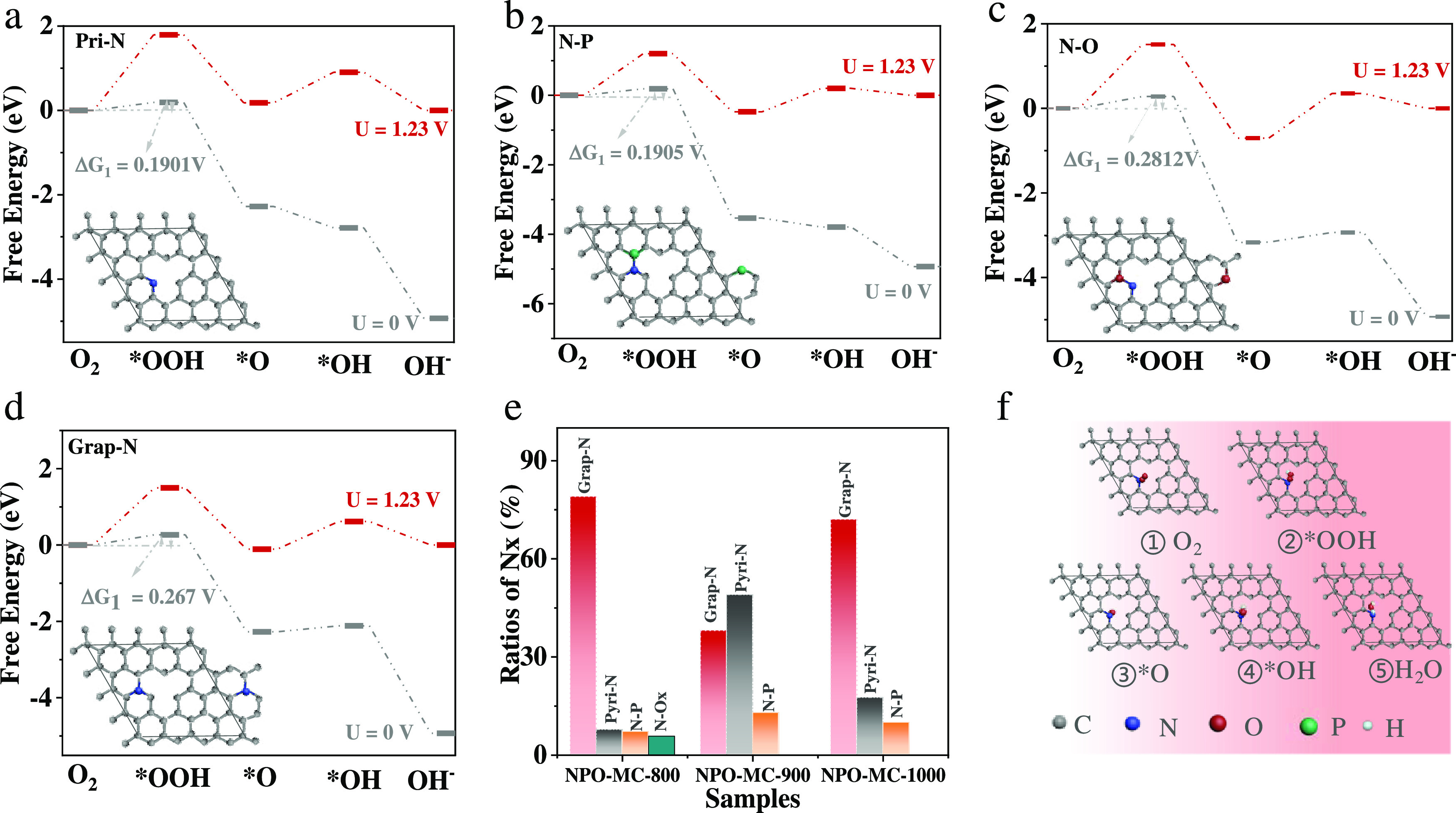
Mechanistic study of different types of N doping. (a–d) Free energy step diagrams of the ORR at the active sites of four models; (e) content of four types of N for NPO-MC-800, NPO-MC-900, and NPO-MC-1000; (f) illustrative ORR pathway on NPO-MC-900 (gray: carbon; blue: nitrogen; red: oxygen; green: phosphorus; white: hydrogen).

To gain an in-depth understanding of the NPO-MC catalyst, DFT calculations were used to expose the active site. Four models of doped carbon were built based on N 1s XPS results (inset Figure 6a–d). The corresponding optimized atomic configurations of doped NPO-MC are shown in Figure S11 (Supporting Information). The adsorption behavior of the above model for the ORR is discussed. Figure 6a–d shows that different types of doped carbon models have different Gibbs free energy changes. It can be concluded that the formation of *OOH intermediates is the rate-limiting step for the ORR of NPO-MC catalysts, which is in line with previous reports.^51,52^ As can be seen from Figure 6a–d, the model has a significant effect on the free energy change. The highest Δ*G* of the first step (O_2_ ∼ *OOH) shows the rate-determining step at *U* = 1.23 V. Therefore, Figure 6b–e and Table S4 give the numerical values of the Gibbs free energy (Δ*G*) changes for the pyri-*N*, grap-*N*, N–P, and N–O models, which are 0.1901, 0.1905, 0.2812, and 0.267 eV at *U* = 0 V, respectively. As a result, the pyri-*N* and N–P-doped NPO-MC possessed lower Δ*G* than that of other forms of doping, indicating the pyri-*N* and N–P-doped NPO-MC have more efficient ORR catalytic activity.^53^ According to the N 1s XPS (Figure 6e), the highest amount of pyri-*N* and N–P were captured in NPO-MC-900, which is a major reason for the excellent catalytic performance of NPO-MC-900. Details of the oxygen reduction reaction for a four-electron pathway are presented in Figure 6f.^54^

Another work has previously been reported on N-doped carbon catalysts. For example, the N-HsGDY is also doped with pyri-*N* and the carbonization temperature is optimal at 900 °C.^55,56^ Therefore, a synergistic effect of pyri-*N* doping, pore structure, and conductivity is probably easier to achieve at 900 °C carbonization. More interestingly, through theoretical calculations, we found that the N–P structure also has a free energy change close to pyri-*N*, which provides ideas for our subsequent research.

## 4 Conclusions

In summary, using HCCP and PEI as N and P sources, a metal-free and bifunctional multipore carbon NPO-MC catalyst was successfully developed by polymerization at room temperature and subsequent carbonization. The oxygen element was added with the process of washing. The obtained NPO-MC-900 catalyst has the characteristics of high specific surface area, porosity, and N,P-codoping. Benefiting from their synergistic interaction, NPO-MC-900 exhibits high electrocatalytic activity and is successfully applied to zinc–air batteries. The highest catalytic activity and lowest free energy barrier of the double doping of pyri-*N* and P can explain the excellent electrocatalytic performance of NPO-MC-900. Meanwhile, our work provides a reference for the preparation of metal-free heteroatom doping for energy applications.

## Supporting Information Available

Sources of raw materials; synthesis, characterization, performance testing, zinc–air cell assembly, and DFT calculations of material, including additional data supplement to experimental data (PDF)
