# Supplementary material for: Advanced and Stable Metal-Free Electrocatalyst for Energy Storage and Conversion: The Structure–Effect Relationship of Heteroatoms in Carbon
Source: ACS Omega. 2023 May 1;8(18):16364–72. doi: 10.1021/acsomega.3c01145 (PMC10173325; doi:10.1021/acsomega.3c01145)
Supplement: Supplementary file 1 — ao3c01145_si_001.pdf [file ao3c01145_si_001.pdf]

Supporting Information

An advanced and stable metal-free electrocatalyst for energy storage and conversion: structure-effect relationship of heteroatoms in carbon

Jingjing Zhang, Kechuang Wan, Pingwen Ming, Bing Li, Cunman Zhang*

Dr. *J. J. Zhang, K. C. Wan,* Prof. *P. W. Ming, B. Li, C. M. Zhang,*

Clean Energy Automotive Engineering Center and School of Automotive Studies, Tongji University, Shanghai 201804, China;

Email: libing210@tongji.edu.cn

Table of Contents:

1. Chemicals.

2. Materials characterization

3. Electrochemical measurements

4. Electrochemical measurements for Zn-air battery

5. DFT Computational

6. Results and Discussion.

7. Figure S1-S9.

8. Tables S1-S6.

5. References.

1. **Chemicals:**

Hexachlorocyclotriphosphazene (HCCP, 98%), Acetonitrile (99.8%), polyethyleneimines (PEI, M.W. 600,99%) and Triethylamine (TEA, AR, 99.0%) were obtationed from aladdin, Ethanol absolute, Deionized water, KOH was purchased from Sinopharm Chemical Reagent Co., Ltd. Nafion perfluorinated resin solution (5 wt. % in mixture of lower aliphatic a lcohols and water, contains 45% water), all the chemicals used in this work were analytical reagents and used without further purification.

The N-C-900 was synthesized as follows: PEI (1.95 g, 0.033 mol) was directly high temperature pyrolysis (under 900 ℃ for 3 hours under N_2 atmosphere) to obtain N-C-900 material.

The NP-900 was synthesized as follows: As HCCP itself contains both N and P elements, it is difficult to prepare catalysts containing only P elements. HCCP (0.00125 mol) was directly high temperature pyrolysis (under 900℃for 3 hours under N_2 atmosphere) to obtain NP-900 material.

The O-C-900 was synthesized as follows: In this experiment, the oxygen was incorporated during the experimental treatment, therefore, we used citric acid as the raw material for the synthesis of O-C. citric acid (0.00125 mol) was directly pyrolysed (under 900℃for 3 hours under N_2 atmosphere) to obtain O-C-900 material.

2. Materials characterization

The as-obtained products were characterized with X-ray diffraction (XRD) (Bruker D8) using Cu Kα radiation (λ = 0.15406 nm), SEM, and energy-dispersive X-ray analysis (Nova Nano SEM 200) operated at an acceleration voltage of 10 kV, and

TEM and HRTEM (JEM-2100, JEOL). Energy-dispersive X-ray spectroscopy was taken at the same time as TEM measurement. Raman spectroscopy (JY-T 643200, France) was performed at ambient temperature with a laser excitation of 514 nm. XPS was performed on a spectrometer from Kratos Axis Ultradld, using Mono Al Kα radiation at a power of 120 W (8 mA, 15 kV). The nitrogen adsorption/desorption data were recorded at the liquid nitrogen temperature (77 K) using a micrometrics apparatus (ASAP 2020 M). The specific surface area was calculated using the BET equation. Microstructure of NPO-MC powder was captured using a microscope (Olympus BX51).

3. Electrochemical measurements

Prior to the surface coating, glassy carbon rotating disk electrode (RDE, 5 mm in diameter) was polished carefully with 1.0, 0.3 and 0.05μm alumina powder, respectively, and rinsed with deionized water, followed by sonicated in ethanol and doubly distilled water successively. All catalysts were prepared by mixing 2 mg of the catalysts in 1 mL of solution containing 480 μL of ethanol, 480 μL of H_2O and 4 μL of 5% Nafion solution, followed by ultrasonication for 30 min to form homogeneous catalysts inks. The obtained catalysts inks were then dropped on the surface of pretreated RDE surface and dried before the electrocatalytic tests, leading to 0.2 and 0.1 mg cm^{-2} loading for the obtained samples and Pt/C, respectively.

All the electrochemical measurements were carried out on WaveDriver 20 (Pine Research Instrumentation) and CHI 660E Potentiostat (CH Instruments) systems equipped with a three-electrode cell. All the measurements were performed at ambient

temperature in a 0.1 M KOH alkaline solution. A glassy carbon electrode (GCE) coated with the catalyst ink was served as the working electrode, a Hg/HgO and Carbon rod wire were used as reference and counter electrode, respectively. Potentials in this work were all referred to the reversible hydrogen electrode (RHE) through the Nernst equation as follows: E (vs. RHE) = E (vs. Hg/HgO) ＋ 0.098 ＋ 0.0591 × pH. Prior to the measurement, a N_2/O_2 flow was used through the electrolyte in the cell for 30 min to saturate it with N_2/O_2. The electrochemical experiments were conducted in O_2-saturated 0.1 M KOH for the oxygen reduction reaction at room temperature. The RDE tests were measured at various rotating speed from 400 to 2500 rpm with a sweep rate of 5 mV s^{-1}. For the ORR at an RDE, the electron transfer number (n) and kinetic current density (J_K) were calculated from the Koutecky-Levich (K-L) equation:

$$\frac{1}{J} = \frac{1}{J_L} + \frac{1}{J_K} = \frac{1}{B\omega^{\frac{1}{2}}} + \frac{1}{J_K}$$

$$B = 0.62nFC_0D_0^{\frac{2}{3}}V^{\frac{1}{6}}$$

where J is the measured current density, J_K and J_L are the kinetic and limiting current densities, ω is the angular velocity of the disk, n is the electron transfer number, F is the Faraday constant (96485 C mol^{-1}), C_0 is the bulk concentration of O_2 (1.2×10^{-6} mol cm^{-3}), D_0 is the diffusion coefficient of O_2 (1.9×10^{-5} cm^2 s^{-1}), and V is the kinematic viscosity of the electrolyte (0.01 cm^2 s^{-1}).

The accelerated durability tests (ADT) of the electrocatalysts were performed in the O_2-saturated 0.1 M KOH electrolyte at room temperature by chronoamperometric responses at a constant potential of 0.6 V for 162000 s.

4. Electrochemical measurements for Zn-air battery

The primary Zn-air batteries were tested in a home-built electrochemical cell. The homogeneous ink was loaded on carbon fiber paper (1 cm^2), with a loading density of 1 mg cm^{-2}. as the air cathode, and a polished Zn foil was used as the anode. A 0.2 M Zn $(OAc)_2$ in 6M KOH aqueous solution was used as the electrolyte. All data were collected from the as-fabricated cell with a Land CT2001A system at room temperature.

For the preparation of NPO-MC-900 electrode, catalyst ink was prepared by mixing 20 mg of catalysts and 4 mL of ethanol, followed by ultrasonication for 30 min to form homogeneous catalysts inks. Then 133 mg of 5% Nafion solution was added to achieve a ratio of 3:1 for the mass of catalyst and Nafion and ultrasonicated for another 30 min. Finally, 3 mg of 60% PTFE emulsion (Hesen) and 150 mg of 0.2% MWCNT slurry (Tuball Batt) were added to achieve a mass ratio of 6% and 1%, respectively. The mixture was ultrasonicated for 1 h before sprayed on a carbon paper (28BC, Sigracet) with an area of 5 × 5 cm^2 at 80 °C using an airbrush (0.3 mm, Baheli). For the preparaton of Pt/C+RuO_2/C electrode, the procedure was almost the same except substituting NPO-MC-900 with 8 mg of Pt/C and 8 mg of RuO_2/C. In addition, the adding amounts of the other components were changed to keep the same mass ratio at the same time. The resulting loadings of NPO-MC-900 and Pt/C+

RuO_2/C electrodes were both 0.9 mg cm^{-2}. After the preparation of the air electrodes, they were cut into discs with a diameter of 14.5 mm using a cutting machine. However, the practical working area for air electrode was 0.7854 cm^2 (1 mm in diameter). Ultimately, air electrode, glass fiber filter and Zn plate were assembled into the cell case for the battery test.

5. Methodology and DFT calculation model

All the theoretical computations data are obtained by the Vienna Ab-initio simulation package (VASP). Electronic exchange and correlation effects are demonstrated by generalized gradient approximation (GGA) and Perdew-Burke-Ernzerhof (PBE) functions, testing the plane wave cutoff and set to 500 eV. Self-consistent field (SCF) tolerance is 1×10^{-6} eV. The Brillouin zone was sampled at a ($4 \times 4 \times 1$) mesh. The Gibbs free energy (G) is calculated by the formula $G = E_{surf} + E_{ZPE} - T\Delta S$, where E_{surf} is the total energy calculated by DFT and E_{ZPE} is the zero-point energy calculated using the vibrational frequency of the adsorbate.

The oxygen reduction reaction (ORR) mechanism involves four steps, with the intermediate products being *OOH, *O, and *OH. This method was developed by Nørskov et. al. Here, the * represents the reaction active sites located at the surface of structure model. Usually, the oxygen atom of reaction intermediates was connected to the active sites, forming a single bond. The largest absorption free energy variation of each step was defined as theoretical values of over-potential (η), which determines the

rate of overall reaction. The four electron ORR pathway could be summarized by the following four elementary steps:

$$* + O_2 + H^+ + e^- \rightarrow * OOH \qquad \Delta G_1$$

$$* OOH + H^+ + e^- \rightarrow * O + H_2O \qquad \Delta G_2$$

$$* O + H^+ + e^- \rightarrow * OH \qquad \Delta G_3$$

$$* OH + H^+ + e^- \rightarrow * H_2O \qquad \Delta G_4$$

6. Results and Discussion:

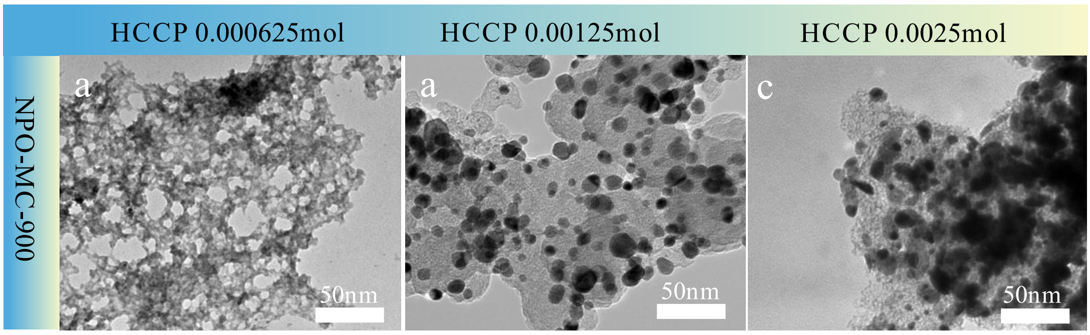

Figure S1. TEM images, a) NPO-MC-0.000625; b) NPO-MC-0.00125; c) NPO-MC-0.0025.

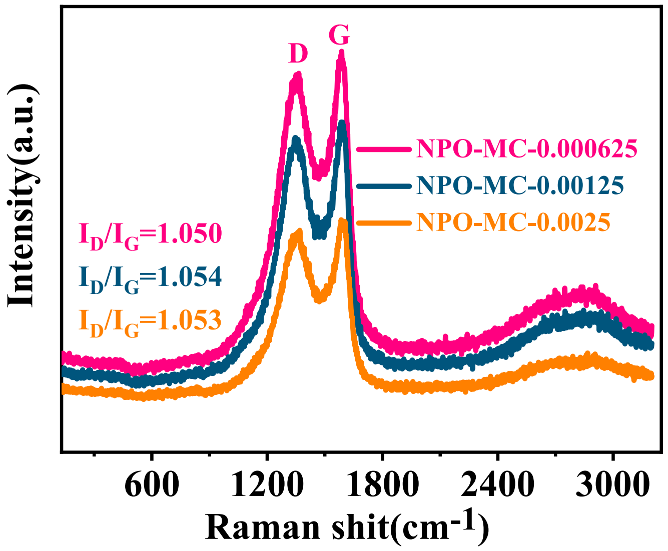

Figure S2. Raman spectra of NPO-MC-0.000625-900, NPO-MC-0.00125-900 and NPO-MC-0.0025-900 samples.

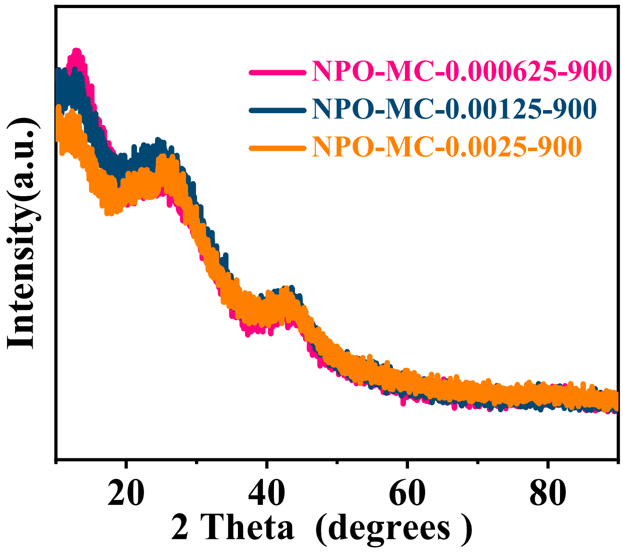

Figure S3. XRD spectra of NPC-0.000625, NPO-MC-0.0025 and NPO-MC-0.00125 samples.

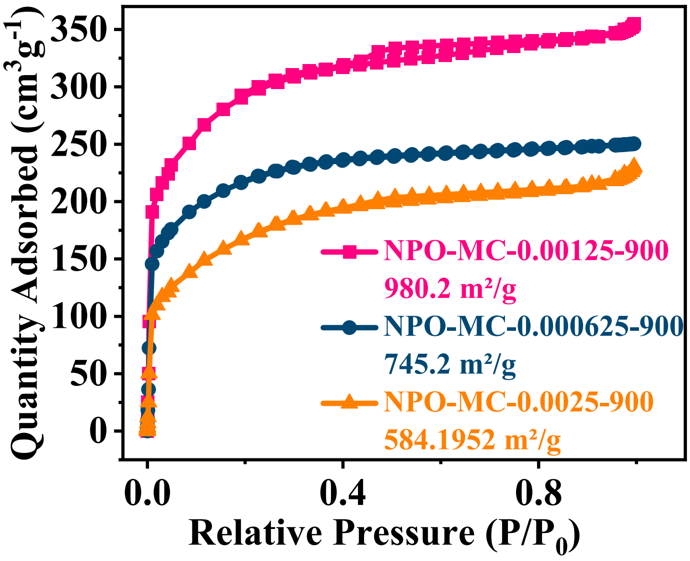

Figure S4. N_2 adsorption-desorption isotherm of NPO-MC-0.000625, NPO-MC-0.0025 and NPO-MC-0.00125 samples.

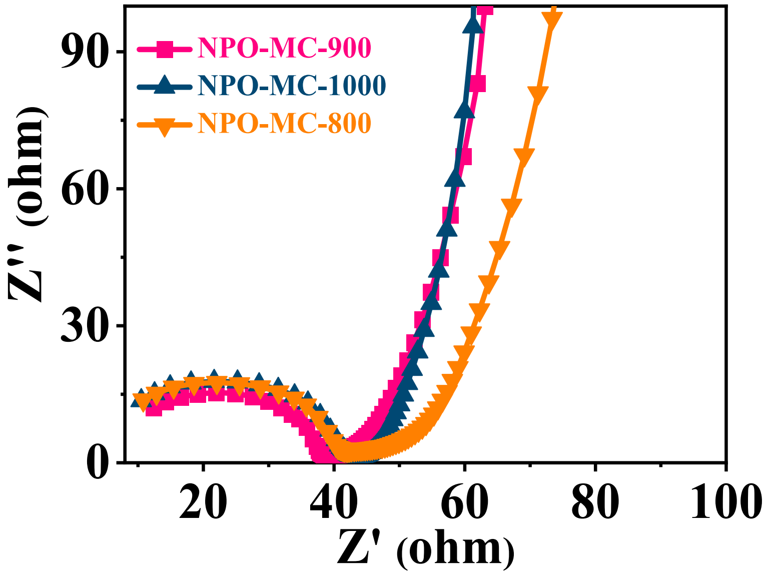

Figure S5. Nyquist plots of NPO-MC-800, NPO-MC-900 and NPO-MC-1000 samples.

Table S1. The atomic ratio from the XPS for the synthesized NPO-MC with the different carbonization temperatures.

NPO-MC 0.00125	800	900	1000
P2p/%	5.12	2.59	1.42
Cl2p/%	0.11	0.16	0.17
C1s/%	67.68	77.92	83.58
N1s/%	8.35	3.44	2.56
O1s/	18.74	15.88	12.26

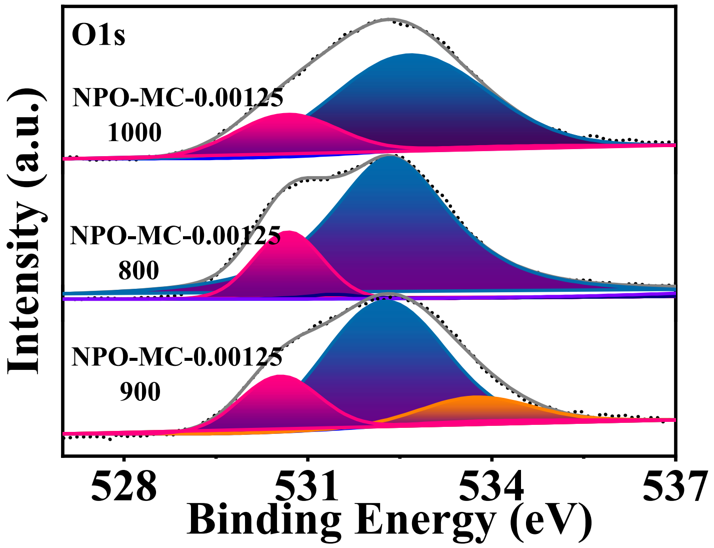

Figure S6. O1s spectrum of NPO-MC-0.00125-1000, NPO-MC-0.00125-900 and NPO-MC-0.00125-800 samples.

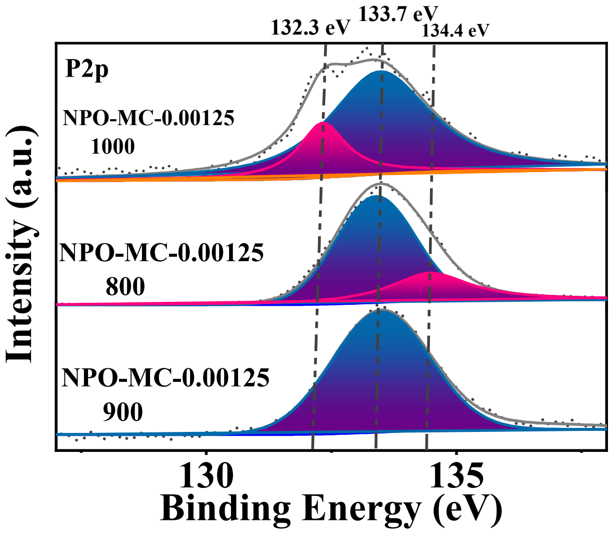

Figure S7. P2p spectrum of NPO-MC-0.00125-1000, NPO-MC-0.00125-900 and NPO-MC-0.00125-800 samples.

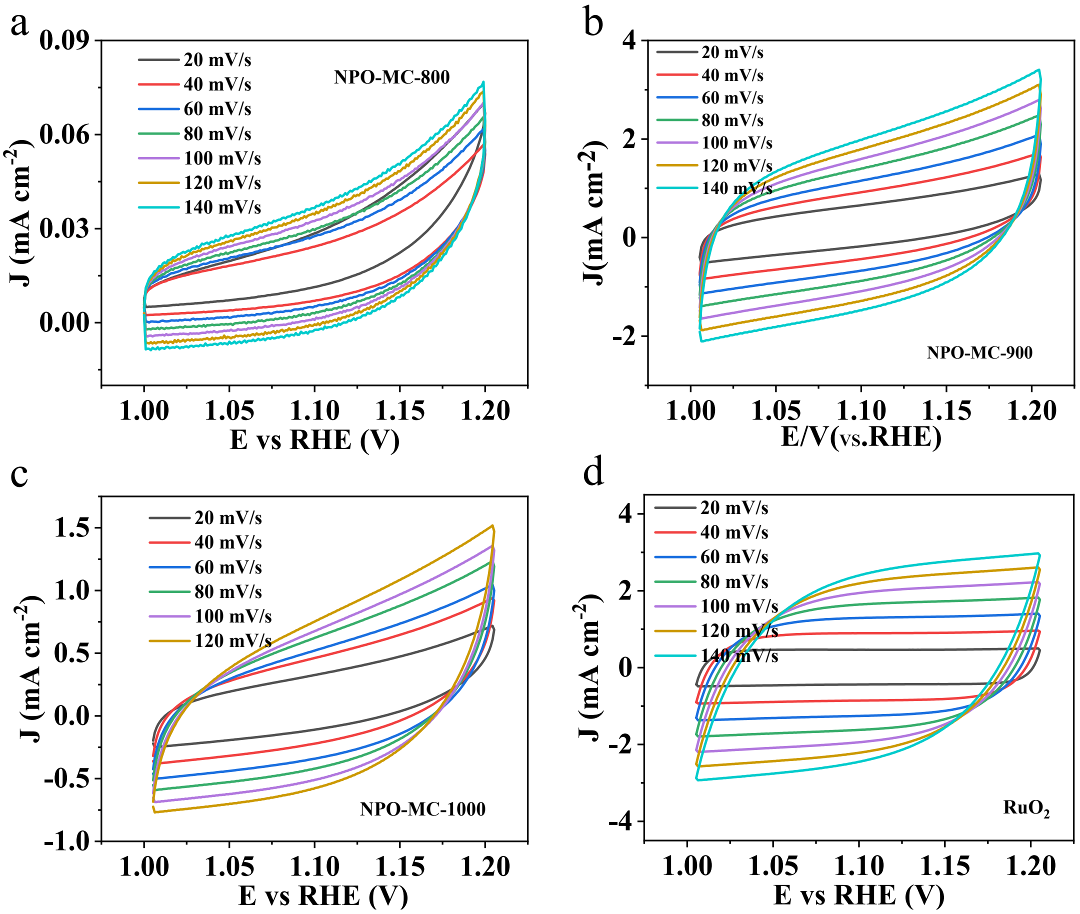

Figure S8. Comparison of CV curves in 0.1 m KOH: a) NPO-MC-800; b) NPO-MC-900; c) NPO-MC-1000; d) RuO_2.

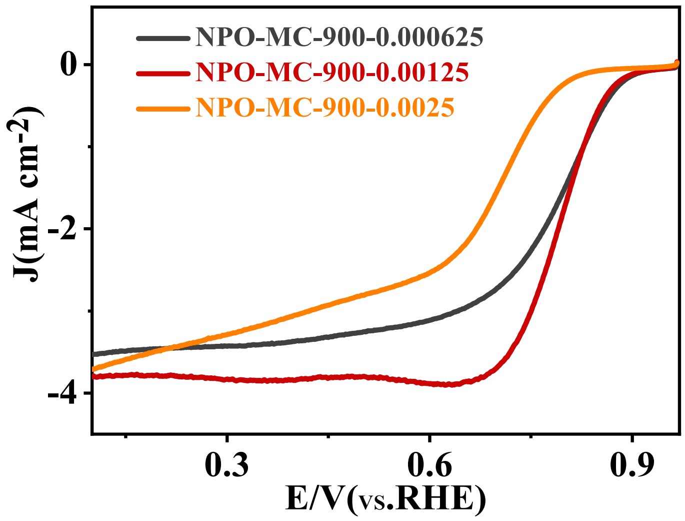

Figure S9. ORR performance of NPO-MC-900-0.000625, NPO-MC-900-0.00125 and NPO-MC-900-0.0025.

Figure S9 shows the ORR catalytic performance of NPO-MC-900-0.000625, NPO-MC-900-0.00125 and NPO-MC-900-0.0025, where the best performance was achieved when the HCCP ratio was 0.00125. Interestingly, the performance decreased when the ratio increased to 0.0025, which may be due to the high N, P content lead to the material's electrical conductivity was reduced. Meanwhile, the low ratio of HCCP leads to low content of N, P, and insufficient active sites, so the performance is not outstanding.

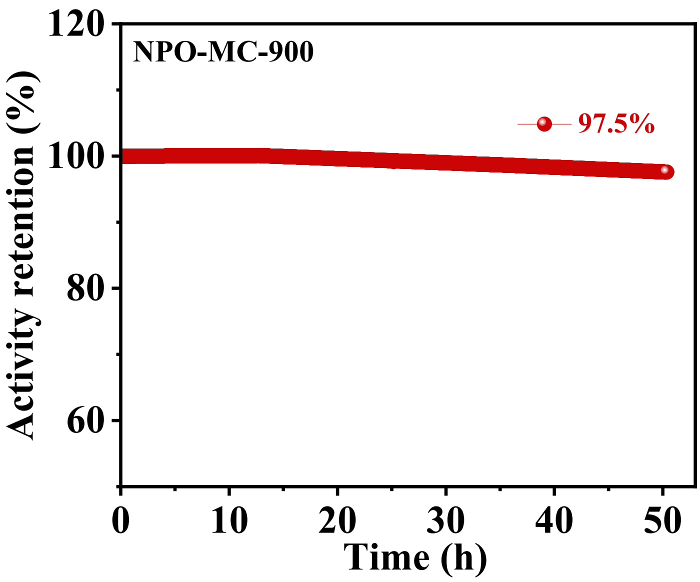

Figure S10. Long-term stability test of the NPO-MC-900 sample carried out under a constant density of 10 mA cm^{-2}.

we performed Chronopotentiometry response measurement (Figure S10) shows that the NPO-MC-900 stably run over 50 h at a current density of 10 mA cm^{-2} without obvious decay in activity, the NPO-MC-900 electrode exhibits 3% activity decay after polarizing for 50h. Therefore, the good stability of NPO-MC-900 is confirmed.

Table S2. Summary of the ORR catalytic performances reported in recent literatures.

Sample	Onset Potential (V)	Half–wave Potential	Ref.
NPO-MC-900	0.91	0.79	This work
20 wt% Pt/C	0.94	0.82	This work
W_2N/WC	0.93	0.81	[1]
Co/Co-N-C	0.86	0.78	[2]
FeCo/Co_2P@NPCF	0.85	0.79	[3]
$NiCo_2S_4$@g-C_3N_4-CNT	0.87	0.76	[4]
Co-POC	–	0.83	[5]
NOGB-800	0.92	0.84	[6]
1-NH_2	–	0.76	[7]
NFPC-1100	0.96	0.85	[8]
NFGD	1.0	0.74	[9]
N 550-GD	0.90	0.75	[10]
Fe-N-GDY	0.94	0.83	[11]
N-CNSP	0.96	0.85	[12]
NPC- “Cs”	~0.93	0.85	[13]

Table S3. Summary of the electrocatalytic performances in Zn–air batteries reported in recent literatures.

Sample	Peak Power Density ($mW\ cm^{-2}$)	Cycling condition and Operating life (h)	Ref.
NPO-MC-900	215	5 mA cm^{-2}, 10 min/cycle for 200 h	This work
20 wt% Pt/C+RuO_2	132	5 mA cm^{-2}, 10 min/cycle for 30 h	This work
NFPC-1100	157	5 mA cm^{-2}, 20 min/cycle for 200 cycles 67 h	[8]
Co/Co-N-C	132	10 mA cm^{-2}, 10 min/cycle for 1000 cycles	[2]
FeCo/Co_2P@NPCF	154	10 mA cm^{-2}, 10 min/cycle for 640 cycles	[3]
Co-POC	78	2 mA cm^{-2}, 20 min/cycle for 237 cycles	[5]
NOGB-800	111.9	10 mA cm^{-2}, cycling for 30 h	[6]
DN-CP@G	135	5 mA cm^{-2}, cycling for 250 cycles	[14]
Co_3HITP_2	164	5 mA cm^{-2}, cycling for 80 h	[15]
Fe-N/P-C-700	133.2	10 mA cm^{-2}, cycling for 40 h	[16]
Ni|MnO/CNF	138.6	10 mA cm^{-2}, 20 min/cycle fo r 350 cycles	[17]
FeNiCo@NC-P	112	10 mA cm^{-2}, 60 min/cycle for 120 cycles	[18]
N-GDY-900	84	5 mA cm^{-2}, cycling for 300 h	[19]
N-CNSP	160	5 mA cm^{-2}, cycling for 150 h	[20]

Table S4. Gibbs free reaction energies for the four-electron transfer during ORR on Pyri-N site, N-P site, N-O site, and Grap-N site.

Elementary reactions	Pyri-N<br>U=0 V	N-P<br>U=0 V	N-O<br>U=0 V	Grap-N<br>U=0 V
$* + O_2 + H^+ + e^- \rightarrow *OOH$	0.1901	0.1905	0.2812	0.267
$*OOH + H^+ + e^- \rightarrow *O + H_2O$	-2.0879	-3.3436	-2.882	-2.005
$*O + H^+ + e^- \rightarrow *OH$	-0.5071	-0.2569	0.2278	0.155
$*OH + H^+ + e^- \rightarrow *H_2O$	-2.1449	-1.139	-1.9946	2.813

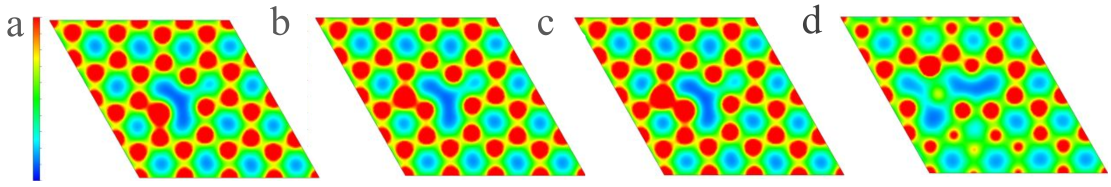

Figure S11. The calculated charge distribution of NPO-MC. (a) Pyri-N; (b) Grap-N; (c) N-O; (d) N-P. The blue and red express zero charge density and maximum charge density.

Table S5. N, P Elemental analysis results of NPO-MC-800, NPO-MC-900 and NPO-MC-1000.

Sample	N (wt %)	P (wt %)
NPO-MC-800	9.23	6.79
NPO-MC-900	8.67	5.5
NPO-MC-1000	6.94	3.21

we conducted elemental analysis tests for NPO-MC-800, NPO-MC-900 and NPO-MC-1000, as shown in Table 1. Among them, NPO-MC-800 has the highest content of N and P elements. However, NPO-MC-1000 has the lowest content of N and P elements, which is probably due to the high loss of N, P elements at high temperatures, NPO-MC-900 has the best performance with moderate content of N and P elements.

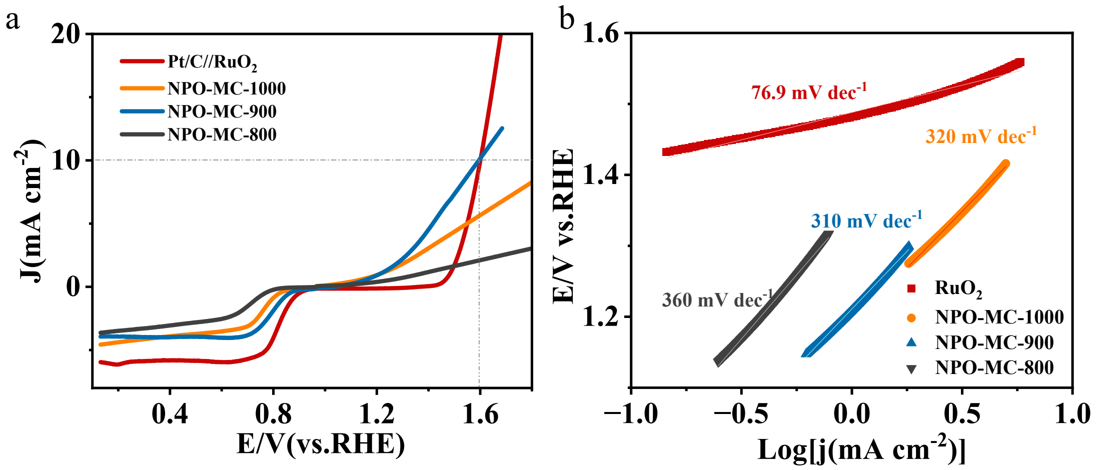

Figure S12. a) ORR and OER performance of four catalysts compared with commercial Pt/C//RuO_2. b) corresponding Tafel plots for RuO_2, NPO-MC-1000, NPO-MC-900, and NPO-MC-800 in O_2-saturated 0.1 m KOH solution at 1600 rpm and 10 mV s^{-1}.

we calculated the Tafel slope of the OER, the Tafel slope in Figure S12b derived from the LSV curves (Figure S12a), Although the Tafel slope of NPO-MC-900 (310 mV dec^{-1}) is larger than that of RuO_2 (76.9 mV dec^{-1}) due to its metal-free property, it is much smaller than those of NPO-MC-1000 (320 mV dec^{-1}) and NPO-MC-800 (360 mV dec^{-1}), proving that an accelerated kinetic process is fulfilled.

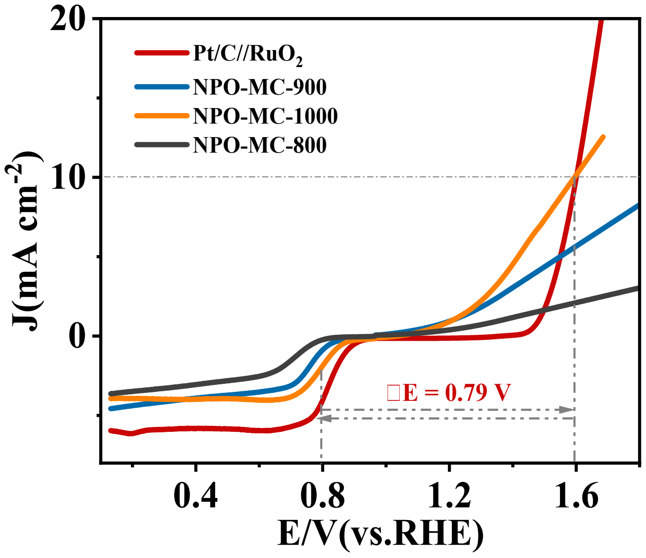

Figure S13. The oxygen electrode activity ($\triangle E=E_{j=10}-E_{1/2}$) of NPO-MC-900.

Table S6. Comparison of oxygen bifunctional performance of NPO-MC-900 and previous excellent catalysts in 0.1 M KOH.

Serial number	Catalyst	$E_{1/2}$ of ORR(V)	$E_{J=10}$ of OER(V)	△E (V)	Ref.
1	CoNC SAC	0.86	1.65	0.79	Sci. Adv. 2022, 8, eabn5091.
2	Ni-N4/GHSs/FN4	0.83	1.62	0.79	Adv. Mater. 2020, 32, 2003134.
3	Co-BTCbipy-700	0.79	1.63	0.84	Energy Storage Mater. 2019, 17, 46
4	Fe_1Co_1-CNF	0.87	1.73	0.86	Nano Energy 2021, 87, 106147.
5	CoSAs-NGST	0.89	1.79	0.9	Adv. Funct. Mater. 2021, 31, 2010472
6	Co-N-PDEB	0.84	1.92	1.08	Appl. Catal. B Environ. 2020, 275, 119107.
7	NPO-MC-900	0.79	1.58	0.79	This work

we calculated Overpotential calculation between ORR and OER processes ΔE ($E_{1/2}$ − $E_{j=\ 10\ mA\ cm^{-2}}$) of NPO-MC-900 as shown in Fig. S13, and compared with reported carbon based bifunctional electrocatalysts (Table S6). Fig. S13 illustrates the ΔE of only 0.79 V of NPO-MC-900 for ORR and OER. In order to intuitively show the performance compared with reported carbon based bifunctional electrocatalysts, a table is list in Table S6. NPO-MC-900 displayed the smallest potential gap (ΔE) of 0.79 V between potential at current density of 10 mA cm^{-2} ($E_{j=10}$) for OER and $E_{1/2}$ for ORR (Fig. S13), which indicates its superior bifunctional catalytic activity that outperforms most of the reported single-site or dual-site electrocatalysts (Table S6).

References:

[1] J. Diao, Y. Qiu, S. Liu, W. Wang, K. Chen, H. Li, W. Yuan, Y. Qu, X. Guo, Interfacial engineering of W2N/WC heterostructures derived from solid-state

synthesis: a highly efficient trifunctional electrocatalyst for ORR, OER, and HER, Adv. Mater. 32 (2020) 1905679.

[2] P. Yu, L. Wang, F. Sun, Y. Xie, X. Liu, J. Ma, X. Wang, C. Tian, J. Li, H. Fu, Co Nanoislands rooted on Co–N–C nanosheets as efficient oxygen electrocatalyst for Zn–air batteries, Adv. Mater. 31 (2019) 1901666.

[3] Q. Shi, Q. Liu, Y. Ma, Z. Fang, Z. Liang, G. Shao, B. Tang, W. Yang, L. Qin, X. Fang, High-performance trifunctional electrocatalysts based on FeCo/Co_2P hybrid nanoparticles for zinc–air battery and self-powered overall water splitting, Adv. Energy Mater. 10 (2020) 1903854.

[4] X. Han, W. Zhang, X. Ma, C. Zhong, N. Zhao, W. Hu, Y. Deng, Identifying the activation of bimetallic sites in $NiCo_2S_4$@g-C_3N_4-CNT hybrid electrocatalysts for synergistic oxygen reduction and evolution, Adv. Mater. 31 (2019) 1808281.

[5] B.-Q. Li, C.-X. Zhao, S. Chen, J.-N. Liu, X. Chen, L. Song, Q. Zhang, Framework-porphyrin-derived single-atom bifunctional oxygen electrocatalysts and their applications in Zn–air batteries, Adv. Mater. 31 (2019) 1900592.

[6] Q. Hu, G. Li, G. Li, X. Liu, B. Zhu, X. Chai, Q. Zhang, J. Liu, C. He, Trifunctional electrocatalysis on dual-doped graphene nanorings–integrated boxes for efficient water splitting and Zn–air batteries, Adv. Energy Mater. 9 (2019), 1803867.

[7] W. Li, S. Xue, S. Watzele, S. Hou, J. Fichtner, A. L. Semrau, L. Zhou, A. Welle, A. S. Bandarenka, R. A. Fischer, Advanced bifunctional oxygen reduction and evolution electrocatalyst derived from surface-mounted metal–organic frameworks, Angew. Chem., Int. Ed. 59 (2020) 5837–5843.

[8] Y. Sun, J. Yang, X. Ding, W. Ji, A. Jaworski, N. Hedin, B. Han, Synergetic contribution of nitrogen and fluorine species in porous carbons as metal-free and

bifunctional oxygen electrocatalysts for zinc–air batteries, Appl. Catal. B. 297 (2021) 120448.

[9] S. Zhang, Y. Cai, H. He, Y. Zhang, R. Liu, H. Cao, M. Wang, J. Liu, G. Zhang, Y. Li, Heteroatom Doped Graphdiyne as Efficient Metal-Free Electrocatalyst for Oxygen Reduction Reaction in Alkaline Medium, J. Mater. Chem. A, 4 (2016) 4738-4744.

[10] R. Liu, H. Liu, Y. Li, Y. Yi, X. Shang, S. Zhang, X. Yu, S. Zhang, H. Cao, G. Zhang, Nitrogen-Doped Graphdiyne as A Metal-Free Catalyst for High-Performance Oxygen Reduction Reactions, Nanoscale, 6 (2014) 11336-11343.

[11] W. Si, Z. Yang, X. Wang, Q. Lv, F. Zhao, X. Li, J. He, Y. Long, J. Gao, C. Huang, Fe, N-Codoped Graphdiyne Displaying Efficient Oxygen Reduction Reaction Activity, ChemSusChem, 12 (2019) 173-178.

[12] L. Zong, W. Wu, S. Liu, H. Yin, Y. Chen, C. Liu, K. Fan, X. Zhao, X. Chen, F. Wang, Metal-Free, Active Nitrogen-Enriched, Efficient Bifunctional Oxygen Electrocatalyst for Ultrastable Zinc-Air Batteries, Energy Storage Mater., 27 (2020) 514-521.

[13] P. Li, H. Jang, J. Zhang, M. Tian, S. Chen, B. Yuan, Z. Wu, X. Liu, J. Cho, A Metal-Free N and P-Codoped Carbon Nanosphere as Bifunctional Electrocatalyst for Rechargeable Zinc-Air Batteries, ChemElectroChem, 6 (2019) 393-397.

[14] C. Hang, J. Zhang, J. Zhu, W. Li, Z. Kou, Y. Huang, In situ exfoliating and generating active sites on graphene nanosheets strongly coupled with carbon fiber toward self-standing bifunctional cathode for rechargeable Zn–air batteries, Adv. Energy Mater. 8 (2018) 1703539.

[15] Y. Lian, W. Yang, C. Zhang, H. Sun, Z. Deng, W. Xu, L. Song, Z. Ouyang, Z. Wang, J. Guo, Y. Peng, Unpaired 3d electrons on atomically dispersed cobalt centres in coordination polymers regulate both oxygen reduction reaction (ORR) activity and

selectivity for use in zinc–air batteries. Angew. Chem., Int. Ed. 59 (2020) 286–294.

[16] Y K. Yuan, D. Lützenkirchen-Hecht, L. Li, L. Shuai, Y. Li, R. Cao, M. Qiu, X. Zhuang, M. K. H. Leung, Y. Chen, U. Scherf, Boosting oxygen reduction of single iron active sites via geometric and electronic engineering: nitrogen and phosphorus dual coordination, J. Am. Chem. Soc. 142 (2020) 2404–2412.

[17] D. Ji, J. Sun, L. Tian, A. Chinnappan, T. Zhang, W. A. D. M. Jayathilaka, R. Gosh, C. Baskar, Q. Zhang, S. Ramakrishna, Engineering of the heterointerface of porous carbon nanofiber–supported nickel and manganese oxide nanoparticle for highly efficient bifunctional oxygen catalysis, Adv. Funct. Mater. 30 (2020), 1910568.

[18] D. Ren, J. Ying, M. Xiao, Y.-P. Deng, J. Ou, J. Zhu, G. Liu, Y. Pei, S. Li, A. M. Jauhar, H. Jin, S. Wang, D. Su, A. Yu, Z. Chen, Hierarchically porous multimetal-based carbon nanorod hybrid as an efficient oxygen catalyst for rechargeable zinc–air batteries, Adv. Funct. Mater. 30 (2020) 1908167.

[19] T. Lu, X. Hu, J. He, R. Li, J. Gao, Q. Lv, Z. Yang, S. Cui, C. Huang, Aqueous/solid state Zn-air batteries based on N doped graphdiyne as efficient metal-free bifunctional catalyst, Nano Energy 85 (2021) 106024.

[20] L. Zong, W. Wu, S. Liu, H. Yin, Y. Chen, C. Liu, K. Fan, X. Zhao, X. Chen, F. Wang, Metal-Free, Active Nitrogen-Enriched, Efficient Bifunctional Oxygen Electrocatalyst for Ultrastable Zinc-Air Batteries, Energy Storage Mater., 27 (2020) 514-521.
